# Feasibility and validity of International Classification of Diseases based case mix indices

**DOI:** 10.1186/1472-6963-6-125

**Published:** 2006-10-06

**Authors:** Che-Ming Yang, William Reinke

**Affiliations:** 1 School of Healthcare Administration, Taipei Medical University, Taipei, Taiwan; 2 School of Public Health, Johns Hopkins University, Baltimore, Maryland, USA

## Abstract

**Background:**

Severity of illness is an omnipresent confounder in health services research. Resource consumption can be applied as a proxy of severity. The most commonly cited hospital resource consumption measure is the case mix index (CMI) and the best-known illustration of the CMI is the Diagnosis Related Group (DRG) CMI used by Medicare in the U.S. For countries that do not have DRG type CMIs, the adjustment for severity has been troublesome for either reimbursement or research purposes. The research objective of this study is to ascertain the construct validity of CMIs derived from International Classification of Diseases (ICD) in comparison with DRG CMI.

**Methods:**

The study population included 551 acute care hospitals in Taiwan and 2,462,006 inpatient reimbursement claims. The 18^th ^version of GROUPER, the Medicare DRG classification software, was applied to Taiwan's 1998 National Health Insurance (NHI) inpatient claim data to derive the Medicare DRG CMI. The same weighting principles were then applied to determine the ICD principal diagnoses and procedures based costliness and length of stay (LOS) CMIs. Further analyses were conducted based on stratifications according to teaching status, accreditation levels, and ownership categories.

**Results:**

The best ICD-based substitute for the DRG costliness CMI (DRGCMI) is the ICD principal diagnosis costliness CMI (ICDCMI-DC) in general and in most categories with Spearman's correlation coefficients ranging from 0.938-0.462. The highest correlation appeared in the non-profit sector. ICD procedure costliness CMI (ICDCMI-PC) outperformed ICDCMI-DC only at the medical center level, which consists of tertiary care hospitals and is more procedure intensive.

**Conclusion:**

The results of our study indicate that an ICD-based CMI can quite fairly approximate the DRGCMI, especially ICDCMI-DC. Therefore, substituting ICDs for DRGs in computing the CMI ought to be feasible and valid in countries that have not implemented DRGs.

## Background

Severity of illness is an omnipresent confounder in any study of patient outcomes or of the effectiveness of medical care. Short of randomization, if the influences of patient characteristics are not controlled to some extent, the impact of the health care intervention of interest may be masked or distorted. Therefore, the ability to measure the heterogeneous severity of illness of hospitals has been recognized as a critical ingredient for improving the management of hospitals and achieving equity in hospital reimbursements under case payment schemes [1].

Severity of illness measurements can be separated into two levels: individual level and hospital level. At the patient level, the severity of each individual patient is assessed independently based upon clinical findings and personal characteristics, which are mostly extracted from medical records; whereas hospital severity of illness describes the aggregate difficulty in the treatment of the diseases presenting themselves at each hospital as compared to other hospitals and most of the information utilized for this purpose comes from administrative data. What we are interested in, from the perspective of healthcare system management and health service research, is how the aggregate severity of illness at the hospital level can be easily derived from the administrative data.

There are various perspectives in the definitions of severity. Resource consumption has been applied as a proxy of severity, especially at the hospital level for various purposes. The most commonly cited and acclaimed for hospital resource consumption measure is the case mix index (CMI). CMI is an administrative data-driven approach based upon patient classifications. The term "case mix" has emerged to reflect the fact that, within a population, individual patients may have a range of risks, and the aggregate outcome reflects the aggregate risks; as a result, case mix is a useful concept when comparing the performance of hospitals and clinicians[2].

The best-known illustration of the CMI as a resource consumption adjustment standard is the Diagnosis Related Group (DRG) CMI used by Medicare in the US. A hospital's DRG CMI measures the complexity of cases treated at that particular hospital relative to the average complexity in a peer group of hospitals[3]. There are two ways to derive a DRG-based CMI: costs and length of stay (LOS)[3]. The DRG-based costliness CMI, which is actually based on charges instead of costs, is used in Medicare's Prospective Payment System[4]. Medicare DRG cost-based CMIs are most well known for their use in estimating resource consumption in prospective payment systems and are presumed to be the criterion standard of resource consumption measures that can be derived from administrative data in our study. The typical equation used is as follows[3]:

DRGCMIh=[∑g(Wg∗Ngh)]/∑gNgh[∑g(Wg∗Ngn)]/∑gNgn
 MathType@MTEF@5@5@+=feaafiart1ev1aaatCvAUfKttLearuWrP9MDH5MBPbIqV92AaeXatLxBI9gBaebbnrfifHhDYfgasaacH8akY=wiFfYdH8Gipec8Eeeu0xXdbba9frFj0=OqFfea0dXdd9vqai=hGuQ8kuc9pgc9s8qqaq=dirpe0xb9q8qiLsFr0=vr0=vr0dc8meaabaqaciaacaGaaeqabaqabeGadaaakeaacqWGebarcqWGsbGucqWGhbWrcqWGdbWqcqWGnbqtcqWGjbqsdaWgaaWcbaGaemiAaGgabeaakiabg2da9maalaaabaGaei4waS1aaabuaeaacqGGOaakcqWGxbWvdaWgaaWcbaGaem4zaCgabeaakiabgEHiQiabd6eaonaaBaaaleaacqWGNbWzcqWGObaAaeqaaOGaeiykaKIaeiyxa0Laei4la8YaaabuaeaacqWGobGtdaWgaaWcbaGaem4zaCMaemiAaGgabeaaaeaacqWGNbWzaeqaniabggHiLdaaleaacqWGNbWzaeqaniabggHiLdaakeaacqGGBbWwdaaeqbqaaiabcIcaOiabdEfaxnaaBaaaleaacqWGNbWzaeqaaOGaey4fIOIaemOta40aaSbaaSqaaiabdEgaNjabd6gaUbqabaGccqGGPaqkcqGGDbqxcqGGVaWldaaeqbqaaiabd6eaonaaBaaaleaacqWGNbWzcqWGUbGBaeqaaaqaaiabdEgaNbqab0GaeyyeIuoaaSqaaiabdEgaNbqab0GaeyyeIuoaaaaaaa@6595@

where

*h *is the hospital for which the index is being calculated; *W*_*g *_is the weight associated with the DRG_*g*_, i.e. the amount of basic case payment before adjustment for each DRG respectively in our study; *N*_*gh *_is the number of cases in the DRG_*g *_in hospital *h*; and *N*_*gn *_is the number of cases in the DRG_*g *_of the entire country.

One of the primary advantages of classifying patients before determining resource consumption as shown in the calculation process above is that the inefficiency of each individual hospital will have less influence on the determination of the index since the weight for each group of patients is based upon the national average instead of a hospital average.

Although Medicare DRGs do not differentiate patients' severity gradients in detail, Medicare DRG CMI has been shown to be able to approximate hospital severity level to a satisfactory degree. For instance, a study conducted in Philadelphia to estimate hospital inefficiency indicated that the addition of the Medicare DRG CMI reduced estimated inefficiency by more than 50% and the incremental effect of a severity of illness variable to an equation with CMI was very small[5]. It was relatively easy for researchers in the US to account for severity from a resource consumption perspective by using Medicare DRG CMI. However, for healthcare researchers around the world who did not have a readymade CMI at hand, the search for a valid substitute has always been a painstaking process. However, the idea of CMI as a resource consumption and severity adjustment standard does not belong exclusively to DRG-like systems. So long as patients can be classified to a certain extent, one can derive a CMI specific to that pertinent classification method.

For the purpose of patient classification, the diagnosis is always the starting point. The standard diagnostic lexicon originated from the International Classification of Diseases (ICD) code, which is applied worldwide. Although the ICD does not take severity of illness or resource consumption into consideration in classifying patients[6], if averaging based upon classification can diminish the variation of treatment patterns, one should be able to apply the ICD to derive the CMI as well. That is to say, we should be able to compute ICD-specific CMIs in a hospital population if the mathematical algorithm is determined and manageable within our computer's computing power. The Medicare DRGs were originally derived by reducing the ICD codes to smaller subsets, and weighted the patients in each subset by cost. Therefore, we can reasonably expect correlations between ICD CMI and DRG CMI to a certain degree.

The research objective of our study is to ascertain the construct validity of CMIs derived from ICD in comparison with DRG CMI. Our hypothesis was CMI derived from the ICD classification should correlate with the Medicare DRG CMI to a certain degree that justifies the application of ICD based CMIs.

Taiwan has been able to accumulate a significant amount of computerized billing files since the initiation of the National Health Insurance (NHI) program in 1995. Therefore, it would also be interesting to determine whether Taiwan's administrative data set can be utilized to provide meaningful information for computing ICD-based CMI in the search for a valid substitute for Medicare CMI in non-American healthcare settings.

## Methods

One of the main thrusts of this study is to apply the US Medicare DRG system to Taiwan's inpatient claims to come up with a Medicare DRG CMI so that it can be compared with the performance of all the other alternative indices. Since the Medicare DRG system has not been formally adopted in Taiwan, we had to import this tool for our research purposes. In this study, the 18th version of GROUPER, the Medicare DRG classification software, was used for assigning each patient a DRG code. GROUPER is a software programmed according to the classification guidelines issued annually by the US Centers for Medicare & Medicaid Services (CMS).

During the process of computing DRG weights, we deviated from the original Medicare design in two aspects. First of all, outliers which are defined as cases with costs more than three standard deviations away from the geometric mean for each DRG are not included in the computation. The purpose of leaving outliers out of the calculations with Medicare is so that they can be reimbursed separately, which does not serve our purpose of approximating severity. Therefore, outliers were included in our calculations. Second, low-volume DRGs are also deleted in the process. Low-volume DRGs are defined as DRGs for which the number of cases is less than a DRG-specific constant based on the mean and standard deviation, and they are chosen so as to guarantee estimate precision of plus or minus 10% of the mean value for a 90% confidence interval around the average cost for each remaining DRG[7]. According to the literature, the practice that DRGs with fewer than 5 to 10 cases are dropped from weighting computation is roughly compatible with the original design[7]. For the sake of simplicity, the cut-off point was set at 5 cases in this study. The DRG costliness CMIs were obtained subsequently.

The next step was to determine the ICD case mix. For the purpose of this study, principal diagnoses and procedures in the claim data were respectively used in the grouping process. In the first approach, the first three digits of the ICD of the principal diagnosis were used to group inpatient cases. Merely adopting the first 3 digits is not a random and spur of the moment practice. It has been applied in other occasions as well. For example, in order to define what constitutes unexpected readmission of the same diagnosis within 14 days of discharge, the Bureau of National Health Insurance (BNHI) mandated if the first three digits of the patient's ICD-9-CM principal diagnoses were the same in the sequential two admissions, the second instance was considered a readmission[8].

For coding diagnosis, there are ICD disease codes from 001 to 999 and supplementary V codes ranging from V01 to V82. Codes with fewer than 5 patients were not used for the calculation. The ICD CMI can be derived through the same equation used for the DRG CMI calculation as follows:

ICDCMIh=[∑g(Wg∗Ngh)]/∑gNgh[∑g(Wg∗Ngn)]/∑gNgn
 MathType@MTEF@5@5@+=feaafiart1ev1aaatCvAUfKttLearuWrP9MDH5MBPbIqV92AaeXatLxBI9gBaebbnrfifHhDYfgasaacH8akY=wiFfYdH8Gipec8Eeeu0xXdbba9frFj0=OqFfea0dXdd9vqai=hGuQ8kuc9pgc9s8qqaq=dirpe0xb9q8qiLsFr0=vr0=vr0dc8meaabaqaciaacaGaaeqabaqabeGadaaakeaacqWGjbqscqWGdbWqcqWGebarcqWGdbWqcqWGnbqtcqWGjbqsdaWgaaWcbaGaemiAaGgabeaakiabg2da9maalaaabaGaei4waS1aaabuaeaacqGGOaakcqWGxbWvdaWgaaWcbaGaem4zaCgabeaakiabgEHiQiabd6eaonaaBaaaleaacqWGNbWzcqWGObaAaeqaaOGaeiykaKIaeiyxa0Laei4la8YaaabuaeaacqWGobGtdaWgaaWcbaGaem4zaCMaemiAaGgabeaaaeaacqWGNbWzaeqaniabggHiLdaaleaacqWGNbWzaeqaniabggHiLdaakeaacqGGBbWwdaaeqbqaaiabcIcaOiabdEfaxnaaBaaaleaacqWGNbWzaeqaaOGaey4fIOIaemOta40aaSbaaSqaaiabdEgaNjabd6gaUbqabaGccqGGPaqkcqGGDbqxcqGGVaWldaaeqbqaaiabd6eaonaaBaaaleaacqWGNbWzcqWGUbGBaeqaaaqaaiabdEgaNbqab0GaeyyeIuoaaSqaaiabdEgaNbqab0GaeyyeIuoaaaaaaa@657B@

where *h *is the hospital for which the index is being calculated; *W*_*g *_is the weight associated with the ICD_*g*_, i.e., national mean for LOS and payments for each three-digit ICD group respectively in our study; *N*_*gh *_is the number of cases in the ICD_*g *_in hospital *h*; *N*_*gn *_is the number of cases in the ICD_*g *_for the entire country.

The same principles applied in computing the DRG weighting can also be used in computing the ICD weighting. Both the ICD principal diagnosis LOS CMI and the ICD principal diagnosis costliness CMI can be calculated. A second possible ICD classification is based on the procedure code only. We only took into account cases that received ICD-coded procedures to come up with a procedure CMI. Both the ICD procedure LOS CMI and the ICD procedure costliness CMI were also calculated.

The administrative data used in this study was the reimbursement claim dataset of the NHI. The BNHI provided the 1998 NHI claim data. The total data set from the BNHI included two files: DD199801_0.DAT and MAB_HOSBSC.DAT. DD199801_0.DAT is the inpatient claim file which records the summary reports of all inpatient discharges islandwide. The total number of entries is 2,462,006. Most variables applied in our study came from this file. MAB_HOSBSC.DAT records the contracting health care providers' characteristics pertinent to the use of health insurance claim processing.

The study population included all hospitals of the general acute care type that have participated in the Department of Health (DOH) accreditation, excluding chronic and specialty hospitals. 551 hospitals were included. Of the 551 hospitals, private hospitals constituted the largest portion of the population, at 73.3%, in terms of ownership. According to Taiwan's Medical Care Act sections 3, 4, and 5, there are three classes of ownership: public hospital, non-profit hospital, and private hospital[9]. According to this classification, public hospitals only constituted 15.4% and non-profit hospitals 11.3% of the total (see Table 1). On average, non-profit hospitals took care of the most patients. Non-profit hospitals as a whole accounted for 42.7% of the inpatient market and the distribution of inpatient volumes among them are less skewed as compared to the other two ownership categories (see Table 1).

The other hospital category is accreditation level. The three basic accreditation levels are medical center, regional hospital and local hospital. Most hospitals were accredited; only 6.5% hospitals were not accredited. Of the total, 2.5% were medical centers; 9.4% regional hospitals; and 81.5% local hospitals. On average, at the individual facility level, medical centers took care of the most patients, whereas local hospitals as a whole comprised 40.6% of the inpatient market. Due to the advantage of accessibility, local hospitals obviously took a larger market share not only in the inpatient department but also in the outpatient department and the ER. However, the inpatient market share percentage is the lowest among the three. That is too say, local hospitals are more outpatient oriented than inpatient oriented. Another feature worth noting is that the distribution of service volumes in each accreditation level is less skewed as a whole compared to all the other classifications (see Table 1).

The other important hospital classification is teaching status. Teaching hospitals constituted 24.5%, whereas non-teaching 75.5% of the total. In addition, teaching hospitals see more patients than non-teaching institutions both in terms of average service volume and market share (see Table 1). All the operational definitions and codings of CMIs and hospital characteristics variables are listed in Table 2.

## Results and discussion

After the construction process, the spectra of all the CMIs were analyzed and are displayed in Table 3. The DRGCMI ranged from 0.32 to 2.83 for the whole hospital population. The ICDCMI-DC ranged from 0.39 to 3.20. The ICDCMI-DL ranged from 0.35 to 4.60. The ICDCMI-PC ranged from 0.27 to 3.06. The ICDCMI-PL ranged from 0.32 to 1.63. Another phenomenon worth noting is that the averages of CMIs are found to be in the following descending order across the board: medical centers, regional hospitals, local hospitals and non-accredited hospitals. The distributions of none of these CMIs were normal according to the one sample Kolmogorov-Smirnov (K-S) test.

The first step of the analyses was conducted based on the entire dataset as a whole, namely all 551 hospitals, without further stratification. The second step of the analyses was performed after stratifying the hospitals according to their teaching status, ownership, and accreditation level. The distribution of the values for each severity variable was not normally distributed. As a result, we had to apply distribution-free statistics, i.e. non-parametric statistics, in our analyses.

Overall, DRGCMI was significantly correlated with all other CMIs. The highest was between DRGCMI and ICD principal diagnosis based costliness CMI (ICDCMI-DC) at 0.688 (see Table 4). The correlations are fairly high in general and the correlations for CMIs based on cost are higher than those involving length of stay. Also, correlations based on diagnosis are higher than those based on procedure.

The second step of the analyses was to stratify the hospitals according to their teaching status, ownership, and accreditation level (see Table 5). In the teaching stratification, the general pattern applies to both teaching and non-teaching hospitals but correlations are higher in the teaching hospitals. With respect to ownership, the general pattern still holds true and correlations are much higher in the non-profit sector. For instance, in the non-profit category, the highest was between DRGCMI and ICD principal diagnosis based costliness CMI (ICDCMI-DC), which reached 0.938. Analysis by accreditation status produces somewhat more ambiguous results. For instance, the highest correlation at the medical center level was found between DRGCMI and ICD procedure based costliness CMI (ICDCMI-PC) at 0.767 instead of between DRGCMI and ICD principal diagnosis based costliness CMI (ICDCMI-DC). But the highest correlations tend to be at medical centers and the lowest at non-accredited institutions. The biggest contrast is found with respect to LOS where the medical center correlation is 0.534, compared to 0.046 at non-accredited institutions.

An overview of subgroup analyses (see Table 5) reveals that diagnosis based indices produce higher correlations than procedure based indices in all cases except for medical centers, and there the differences are slight and probably non-significant. With this rather unimportant exception, it is noteworthy that the correlations for ICD principal diagnosis based costliness CMI (ICDCMI-DC) are always higher than any of the others. In the LOS analyses, the diagnosis based indices generally produce higher correlations than the procedure based indices with three exceptions: teaching hospitals, regional hospitals, and non-accredited hospitals.

In summary, generally speaking, the best substitute for DRGCMI is ICD principal diagnosis based costliness CMI (ICDCMI-DC). After stratification, the best substitute for DRGCMI in both the teaching and non-teaching categories is still ICD principal diagnosis based costliness CMI (ICDCMI-DC). The same applies to the public sector, the non-profit sector, and the private sector. For medical centers, the best substitute for DRGCMI is ICD procedure based costliness CMI (ICDCMI-PC). For regional, local and non-accredited hospitals, the best substitute for DRGCMI is still ICD principal diagnosis based costliness CMI (ICDCMI-DC).

In our final ranking, correlation coefficients above 0.8 were deemed as good, between 0.8 and 0.5 as fair, and below 0.5 as poor. The reasoning behind this classification is that a correlation coefficient of 0.5 only explains 25% of the variation. Any approximation catching less than 25% of the variation can hardly be described as having good performance. On the other hand, when assessing multicollinearity for regression analyses, a frequent practice is to examine the bivariate correlations among the independent variables, looking for coefficients of about 0.8 or larger[10]. As a result, if the correlation coefficient was higher than 0.8, we were inclined to conclude that the measure was a good proxy or substitute for the well-established standard which was DRGCMI in this instance.

If we simply look at the sample hospitals as a whole, the best alternative to DRGCMI was ICD principal diagnosis based costliness CMI (ICDCMI-DC) as indicated in Table 4. As the performance assessment standard indicated, all of the ICD-based CMIs were shown to be fairly well correlated with DRGCMI. However, among non-DRG methods, the ICD principal diagnosis based costliness CMI (ICDCMI-DC) appears to be the best among those fair substitutes for the DRGCMI. In the comparison between the ICD principal diagnosis based CMIs and ICD procedure based CMIs, the ICD principal diagnosis based CMIs perform better in terms of correlation coefficients under most circumstances. Since not all inpatient admissions have been accorded procedure codes, for instance, most medical inpatients will not have procedure codings, that's probably why ICD principal diagnosis based CMIs correlate better with DRGCMI. The other finding worth noting is that cost based indices performs better than LOS based indices. Since our gold standard, DRGCMI, is in effect a cost based index, it is reasonable that it correlates better with cost based CMIs than LOS based CMIs.

On the other hand, the performances of all indices could also be compared from different angles through stratification. After stratification, all in all, ICDCMIs are mostly fair substitutes for DRGCMI, and ICD principal diagnosis based costliness CMI (ICDCMI-DC) still appears to be the best among those non-DRG based CMIs. ICD principal diagnosis based costliness CMI (ICDCMI-DC) even had good performance in the non-profit sector. Non-profit hospitals have the largest market share in Taiwan. The patient profiles of the non-profit sector might be more representative of the general patient population, which adds to the validity of substituting ICDCMI-DC for DRGCMI. ICD procedure based costliness CMI (ICDCMI-PC) only outperforms ICD principal diagnosis based costliness CMI (ICDCMI-DC) in medical centers. Medical centers are supposed to be tertiary care hospitals, and as a result likely to be more procedure intensive. So it makes sense that we can differentiate severity gradient better among medical centers from the procedure perspective in our study. All in all, the correlations are lowest for the non-accredited hospitals. This is likely due to the fact that non-accredited hospitals comply with fewer standards and there is less consistency across their operations. However, the different performances of the same method in various strata indicate the possibility of the irony that there is no panacea in our search. We simply have to apply the most appropriate method under given circumstances.

The logic of our study can be corroborated by the development of the ICD-9-based Illness Severity Score (ICISS). The ICISS was developed from the Agency for Health Care Policy Research's Health Care Utilization Project database and is used to predict hospital survival, hospital length of stay, and hospital charges of injured patients admitted to University of North Carolina hospitals. Rutlege et al. performed a retrospective study of 9,483 trauma patients at University of North Carolina hospitals to compare the outcome predictions of ICISS with those of the Medicare DRG and the 3 M product All Patient Refined DRG (APR-DRG) systems[11]. The APR-DRG is considered to be an enhancement of DRG structure by adding four severity subclasses to each DRG[12]. The ICISS proved to be superior to both the DRG and APR-DRG in predicting all three.

The foremost limitation of this study is the reliability of coding by hospitals. We relied heavily on the administrative data submitted by hospitals to the BNHI. There are several potential problems with the ICD coding. First of all, hospitals might upcode intentionally to gain reimbursement advantages. Second, hospital staff might not be trained sufficiently well to code correctly. Third, some of the data we needed in our study were not mandated by the government to be coded. For instance, the procedure code is not required to be recorded other than for some specific procedures. Most hospitals tend to code only major procedures, such as operations. Therefore, we might have underestimated the procedure intensity in many facilities. Nonetheless, administrative coding data are the most convenient health services research data sources. The validity of applying administrative coding data has been established to a certain extent in various settings. For instance, a Canadian study indicated that administrative data generally agree with patient chart data for recording of comorbidities in calculating the Charlson index, κ value ranged from 0.87-0.34, although comorbidities tend to be under-reported in administrative data [13].

Applying Medicare DRGs to a non-American system also has inherent problems that limit the generalizability of our results. First of all, Medicare DRGs primarily apply to the aged, disabled, and patients with end stage renal disease (ESRD). If we simply based our analyses on the subset of Taiwanese patients that were aged, disabled or with ESRD, we might be able to anticipate stronger correlations in our comparisons. However, since our goal was to come up with a generic measure at the hospital level and Medicare DRG is merely the tool we chose to validate our approach, we opted for not leaving out any groups of inpatients so as to achieve the objective of finding a solution that can represent the whole hospital. Secondly, the reality that the different versions we used cannot be 100% matched poses another threat to reliability. For instance, Taiwan was still using the 1992 ICD-9-CM for reimbursement coding in 1998, and we had to apply the 2000 GROUPER to a 1998 Taiwan data set. However, in dealing with a huge data set in an international comparative study, we can only try to safeguard the reliability as much as possible. If all the above-mentioned coding problems can be more appropriately dealt with in future studies, it would be all for the better.

Another limitation warranting further study is the substitution of cost for charge in our analyses. As indicated above, this study basically followed Medicare's approach in coming up with costliness CMI, i.e., Medicare uses charges to estimate the severity of the case mix instead of costs, even though it operates under the guise of a cost-based system. Correlations between cost – and charge-based weights had been proved to be very high in numerous studies. It has been said that within each year, the correlation between these two bases exceeds 0.997[14]. However, we still cannot ignore the fact that charge is not cost per se. That is exactly why under the same reimbursement scheme, assuming all other factors being equal, some hospitals can have profits and some cannot. Medicare's current approach only accounts for the changes of case mix in reimbursement adjustment, yet fails to take into account real cost fluctuations. Therefore, if we were able to construct CMIs based on real cost, the CMI-based adjustment should be much more realistic and equitable. Although it is quite difficult to acquire cost data, it is certainly a better approach worthy of acknowledging.

## Conclusion

This study does not advocate that purely ICD-based CMIs can replace all the other more sophisticated systems, such as the Medicare DRG system or 3M's APR-DRG. However, our findings certainly strengthen the legitimacy of applying the ICD system plainly for the purposes of establishing a resource consumption index to approximate severity. The results of our study indicate that an ICD-based CMI can quite fairly approximate the Medicare DRG CMI, especially the ICD principal diagnosis based costliness CMI (ICDCMI-DC). Therefore, the idea of substituting ICDs for DRGs in computing the CMI ought to be feasible and valid in countries that have not implemented DRGs.

## Abbreviations

ACC: Accreditation status

APR-DRG: All Patient Refined DRG

BNHI: Bureau of National Health Insurance

CMI: case mix index

CMS: Centers for Medicare & Medicaid Services

DRG: Diagnosis Related Group

DRGCMI: DRG costliness CMI

DOH: Department of Health

EDU: Teaching status

ERSD: end stage renal disease

ICD: International Classification of Diseases

ICDCMI-DC: ICD principal diagnosis costliness CMI

ICDCMI-DL: ICD principal diagnosis LOS CMI

ICDCMI-PC: ICD procedure costliness CMI

ICDCMI-PL: ICD procedure LOS CMI

ICISS: ICD-9-based Illness Severity Score

K-S: Kolmogorov-Smirnov

LOS: length of stay

NHI: National Health Insurance

OWNER: Ownership

## Declaration of competing interests

The author(s) declare that they have no competing interests.

## Authors' contributions

Che-Ming Yang conceived of and carried out the study, and drafted the manuscript. William Reinke participated in the design and statistical analyses of the study, and helped draft the manuscript. All authors read and approved the final manuscript.

## Pre-publication history

The pre-publication history for this paper can be accessed here:



## Figures and Tables

**Table 1:** Distribution of hospitals according to relevant categories and their respective service volumes and market shares in 1998

		Outpatient		ER visits		Inpatient			
		
	Percentage of the hospital population	Average volume per hospital	Market share of each category	Average volume per hospital	Market share of each category	Average volume per hospital	Market share of each category	Volume variation	Skewness of volume distribution
Ownership									
Public hospital	15.4%	232045	25.2%	14227	24.8%	7177	25.4%	1–75082	4.038
Non-profit hospital	11.3%	406864	35.9%	29123	41.0%	14855	42.7%	156–131594	3.718
Private hospital	73.3%	71609	38.9%	3928	34.2%	1797	31.9%	1–30142	5.064
Accreditation level									
Medical center	2.5%	1245663	24.2%	65486	20.4%	39107	24.7%	9684–131594	1.949
Regional hospital	9.4%	434766	25.3%	33965	31.7%	16712	31.8%	384–42542	1.228
Local hospital	81.5%	82680	47.1%	5039	46.2%	2171	40.6%	2–14520	2.406
Non-accredited hospital	6.5%	37129	3.4%	1166	1.7%	944	2.9%	1–2984	2.056
Teaching status									
Non-teaching	75.5%	57095	32.9%	2755	25.5%	1232	23.1%	1–14060	3.849
Teaching	24.5%	408778	67.1%	28251	74.5%	12669	76.9%	15–131594	4.110
Location factor									
Rural	67.8%	107987	53.0%	7144	56.3%	3460	56.2%	1–131594	9.459
Urban	32.2%	188413	47.0%	10902	43.7%	6008	43.8%	5–75082	3.389

* Data source: SER87F.TXT from DOH, Taiwan. All volumes were counted in terms of numbers of admissions.

**Table 2:** Definition of Variables

CMI variables		
Severity of illness index	Abbreviation	Definition

DRG costliness CMI	DRGCMI	CMI based on payments for DRGs
ICD principal diagnosis costliness CMI	ICDCMI-DC	CMI based on payment under ICD principal diagnosis groupings
ICD principal diagnosis LOS CMI	ICDCMI-DL	CMI based on LOS under ICD principal diagnosis groupings
ICD procedure costliness CMI	ICDCMI-PC	CMI based on payment under ICD procedure code groupings.
ICD procedure LOS CMI	ICDCMI-PL	CMI based on LOS under ICD procedure code groupings

Hospital variables		

Accreditation status	ACC	Medical center (ACC = 1)
		Regional hospital (ACC = 2)
		Local hospital (ACC = 3)
		Non-accredited (ACC = 4)
Ownership	OWNER	Public (OWNER = 1)
		Non-profit (OWNER = 2)
		Private (OWNER = 3)
Teaching status	EDU	Teaching (EDU = 1)
		Non-teaching (EDU = 0)

**Table 3:** Descriptive statistics of CMIs

	Minimum	Maximum	Mean	Std. Deviation	Skewness
DRGCMI					

All hospitals	0.32	2.83	0.8160	0.2345	2.319
Medical center	0.85	1.4	1.1892	0.1427	-0.895
Regional hospital	0.71	1.52	0.9937	0.1436	0.978
Local hospital	0.39	2.83	0.7891	0.2216	3.157
Non-accredited hospital	0.32	1.88	0.7499	0.2853	1.87

ICDCMI-DC					

All hospitals	0.39	3.20	0.8399	0.2321	3.112
Medical center	1.05	1.36	1.1822	0.09667	0.305
Regional hospital	0.67	1.29	0.9947	0.0971	-0.17
Local hospital	0.39	2.66	0.8162	0.2043	2.336
Non-accredited hospital	0.39	3.2	0.7788	0.4492	4.668

ICDCMI-DL					

All hospitals	0.35	4.60	0.9129	0.2535	5.777
Medical center	0.94	1.17	1.084	0.0694	-0.534
Regional hospital	0.66	1.33	1.019	0.1104	0.097
Local hospital	0.35	4.6	0.9046	0.2671	6.177
Non-accredited hospital	0.36	1.21	0.7969	0.1831	-0.284

ICDCMI-PC					

All hospitals	0.27	3.06	0.7964	0.2181	3.727
Medical center	1.03	1.35	1.1987	0.1144	-0.148
Regional hospital	0.68	1.42	0.9896	0.1414	0.673
Local hospital	0.27	3.06	0.7695	0.2091	5.081
Non-accredited hospital	0.47	1.01	0.6974	0.1185	1.126

ICDCMI-PL					

All hospitals	0.32	1.63	0.9273	0.1550	-1.020
Medical center	0.97	1.12	1.0526	0.0499	-0.234
Regional hospital	0.73	1.09	1.0001	0.0631	-2.178
Local hospital	0.32	1.63	0.9201	0.1588	-0.798
Non-accredited hospital	0.38	1.06	0.8625	0.1699	-1.765

**Table 4:** Spearman's correlations between DRG CMI and other CMIs

	ICDCMI-DC	ICDCMI-DL	ICDCMI-PC	ICDCMI-PL
R	0.688	0.586	0.605	0.444
P	< 0.001	< 0.001	< 0.001	< 0.001

**Table 5:** Spearman's correlations between DRG CMI and the other CMIs according to hospital characteristics

			DRGCMI	ICDCMI-DC	ICDCMI-DL	ICDCMI-PC	ICDCMI-PL
EDU							

0	DRGCMI	R	1.000	0.608	0.504	0.507	0.388
		P		< 0.001	< 0.001	< 0.001	< 0.001
		N	416	416	416	416	416
1	DRGCMI	R	1.000	0.660	0.524	0.620	0.550
		P		< 0.001	< 0.001	< 0.001	< 0.001
		N	135	135	135	135	135

OWNER							

1	DRGCMI	R	1.000	0.462	0.352	0.267	0.292
		P		< 0.001	0.001	0.014	0.007
		N	85	85	85	85	85
2	DRGCMI	R	1.000	0.938	0.741	0.852	0.610
		P		< 0.001	< 0.001	< 0.001	< 0.001
		N	62	62	62	62	62
3	DRGCMI	R	1.000	0.620	0.506	0.586	0.412
		P		< 0.001	< 0.001	< 0.001	< 0.001
		N	404	404	404	404	404

ACC							

1	DRGCMI	R	1.000	0.732	0.534	0.767	0.490
		P		0.003	0.049	0.001	0.075
		N	14	14	14	14	14
2	DRGCMI	R	1.000	0.563	0.298	0.467	0.423
		P		< 0.001	0.032	< 0.001	0.002
		N	52	52	52	52	52
3	DRGCMI	R	1.000	0.643	0.562	0.523	0.364
		P		< 0.001	< 0.001	< 0.001	< 0.001
		N	449	449	449	449	449
4	DRGCMI	R	1.000	0.486	0.046	0.437	0.096
		P		0.003	0.788	0.008	0.578
		N	36	36	36	36	36
